# Efficacy of methylprednisolone in very early systemic sclerosis: results of the ‘Hit Hard and Early’ randomized controlled trial

**DOI:** 10.1093/rheumatology/keae156

**Published:** 2024-03-29

**Authors:** Brigit E Kersten, Jacqueline M J Lemmers, Amber Vanhaecke, Arthiha Velauthapillai, Wieneke M T van den Hombergh, Frank H J van den Hoogen, Cornelia H M van den Ende, Vanessa Smith, Madelon C Vonk

**Affiliations:** Department of Rheumatology, Radboud University Medical Center, Nijmegen, The Netherlands; Department of Rheumatology, Radboud University Medical Center, Nijmegen, The Netherlands; Department of Rheumatology, University Hospital Gent, Gent, Belgium; Department of Rheumatology, Radboud University Medical Center, Nijmegen, The Netherlands; Department of Rheumatology, Radboud University Medical Center, Nijmegen, The Netherlands; Department of Rheumatology, Radboud University Medical Center, Nijmegen, The Netherlands; Department of Rheumatology, Radboud University Medical Center, Nijmegen, The Netherlands; Department of Rheumatology, University Hospital Gent, Gent, Belgium; Department of Rheumatology, Radboud University Medical Center, Nijmegen, The Netherlands

**Keywords:** scleroderma and related disorders, Raynaud’s syndrome, immunosuppressants, interventional studies, inflammation

## Abstract

**Objective:**

We hypothesized that glucocorticoids would induce remission in very early systemic sclerosis (SSc) patients by inhibition of inflammation driving the disease. We examined the efficacy and safety of methylprednisolone in very early SSc.

**Methods:**

In this trial adults with puffy fingers for less than 3 years, specific auto-antibodies and meeting the Very Early Diagnosis of Systemic Sclerosis criteria were randomly assigned (2:1) to methylprednisolone 1000 mg i.v. or placebo for three consecutive days three times with monthly intervals. The primary end point was nailfold capillary density at week 12. Capillary density at 52 weeks, number of megacapillaries and patient-reported outcomes were secondary outcomes. In addition, we assessed disease progression and lung function decline over 52 weeks. We used linear regression analyses adjusted for baseline values and stratification variables to estimate differences between groups.

**Results:**

Between February 2017 and February 2021, 87 patients were screened, of whom 30 (70% female, median [interquartile range, IQR] age 52.9 [40.8–60.8] years, median [IQR] disease duration 11.4 [4.6–18.6] months) were randomly assigned to methylprednisolone (*n* = 21) or placebo (*n* = 9). We found no difference in nailfold capillary density at 12 weeks (−0.5 [95% CI: −1.1, 0.2]) nor in any of the secondary outcomes. Eleven (37%) patients showed disease progression during 1 year follow-up, and seven (23%) patients had a relevant pulmonary function decline. No serious adverse events were reported.

**Conclusion:**

No clinically relevant effect of short-term methylprednisolone in patients with very early SSc was observed. A substantial proportion of patients showed disease progression.

**Trial registration:**

clinicaltrials.gov, NCT03059979.

Rheumatology key messages

- Methylprednisolone is ineffective in stopping disease progression in very early systemic sclerosis.
- A third of very early systemic sclerosis patients showed disease progression after 1 year.

## Introduction

Systemic sclerosis (SSc) is a systemic autoimmune disease of unknown aetiology, characterized by inflammation, vasculopathy and fibrosis of the skin and internal organs. Organ involvement occurs often in the first 3 years after onset of the disease. The extent and degree of organ involvement is linked to mortality, which is increased in patients with SSc with a 5-year mortality rate of 9–16%. Interstitial lung disease and pulmonary arterial hypertension are the main causes of death in SSc [1].

Dysfunction of the vasculature, activation of fibroblasts and dysregulation of the immune system are considered to be the main characteristic pathogenic features of SSc, eventually leading to macro- and microvascular remodelling and finally to fibrosis. Once the fibrosis process has been initiated, it seems to become self-sustaining [2]. Accumulating evidence indicates that, in the very early course of the disease, inflammatory mechanisms drive SSc vasculopathy and fibrosis. In very early SSc there are perivascular and tissue inflammatory infiltrates consisting of monocytes, macrophages and CD4^+^ T lymphocytes [3–5].

Currently, treatment in SSc is initiated when either skin or organ fibrosis becomes apparent and progressive. Treatment options for SSc consist of immunomodulation such as methotrexate, cyclophosphamide or mycophenolate mofetil. In selected patients with poor prognosis autologous stem cell transplantation is considered. Although autologous stem cell transplantation increases the event-free survival in patients with SSc, this treatment is hampered by a relative high treatment-related mortality [6–9].

As for immunomodulating drugs, glucocorticoids are among the most potent and rapidly acting agents of which potential risks and adverse events are well known. Glucocorticoids have an immediate and profound effect on the cellular functions of monocytes, macrophages, neutrophils and endothelial cells mediating the trafficking of leucocytes and affect both T and B lymphocytes, and therefore seem to influence all pathogenic hallmarks involved in early SSc [10]. A systematic review of glucocorticoid monotherapy in SSc showed conflicting results on skin and lung involvement, but positive effects on myopathy [11]. Recently data of the prematurely terminated PRedSS trial was published in which treatment with oral prednisone 0.3 mg/kg during 6 months was compared with placebo in early diffuse cutaneous SSc (dcSSc) [12]. Although the results must be interpreted with caution, patients receiving prednisone had significantly less pain, anxiety and hopelessness [13]. These findings support our hypothesis that glucocorticosteroids have a positive influence on the inflammatory process in SSc considering that pain in dcSSc through musculoskeletal and skin involvement has an inflammatory basis. Studies on high dosage glucocorticoids in SSc are not available. To date, there are no studies that target patients with very early SSc prior to the development of fibrosis. Since in early stages of SSc the pathophysiology is mainly inflammatory driven, treatment with anti-inflammatory drugs may be of key importance to stop progression and improve outcome [2, 14, 15]. However, in order to treat patients early we need to diagnose patients early, which is facilitated by, among others, the Very Early Diagnosis Of Systemic Sclerosis, the VEDOSS criteria [16–19]. Recent research has shown that more than half of the patients fulfilling the VEDOSS criteria have disease progression within 5 years of follow-up, but application of these criteria results in a heterogeneous mixture of patients with both early and longstanding mild disease [20, 21].

Selecting objective outcome measures for intervention trials in patients with very early disease can be challenging because the commonly used outcome measures in SSc (such as modified Rodnan skin score and results of pulmonary function testing) are normal or not sensitive enough to detect changes in these patients. The microcirculation, as can be visualized with nailfold capillary microscopy (NCM), has been shown to be sensitive to change after autologous stem cell transplantation. In recent observational research capillary density was found to be increased 3 months after this treatment, with a durable effect up to 24 months post-intervention [22, 23]. Therefore, changes in capillary density could be an objective derivative outcome measurement for treatment response in very early SSc [22]. Furthermore, capillary density has been attested to be the most reliable inter-rater capillaroscopic parameter [24].

In this proof-of-concept study we hypothesize that by inhibition of the inflammatory process, treatment with intravenously administered glucocorticoids will induce remission in patients with very early SSc. We performed a double-blind, randomized, placebo controlled, multicentre trial in very early SSc patients, to analyse the efficacy and safety of high dose glucocorticoids therapy on nailfold capillary density [25].

## Methods

The Hit Hard and Early trial is a double-blind, randomized, placebo-controlled, multicentre trial [25]. The trial was performed between February 2017 and February 2021 at the Department of Rheumatology of Radboud University Medical Center (Radboudumc) in Nijmegen, The Netherlands and the Department of Rheumatology of the University Hospital Gent in Belgium. The protocol of our study was published previously [25]. During the trial some adjustments to the published study protocol were made. We widened the inclusion criteria from anticentromere and anti-topoisomerase autoantibody to SSc associated autoantibodies. The exclusion criteria for pulmonary organ involvement were specified as diffusing capacity for carbon monoxide <80% predicted and vital capacity <70% predicted caused by significant interstitial lung disease associated with SSc. In addition we changed the trial from monocentre to multicentre. All amendments were approved by the specific medical ethical committees. The study is registered with clinicaltrials.gov, number NCT03059979. Data were monitored by two independent organizations. A data safety monitoring board with two rheumatologists and one epidemiologist was initiated. Two patients were involved in designing of the study. Approval for the study was given by country specific medical ethical committees; the Commissie Mensgebonden Onderzoek (CMO) Arnhem–Nijmegen (METC no. 2015-2097) for the Netherlands, and the Commissie Voor Medische Ethiek University Hospital Gent (EC UZG 2019/047) for Belgium. This is in accordance with Good Clinical Practice and the Declaration of Helsinki.

### Patients

Patients were screened for eligibility at the departments of rheumatology of the Radboudumc and University Hospital Gent. Physicians of referring hospitals were informed about the study through written materials and oral presentations at their local hospital and at national conferences. Possible eligible patients were informed by their physician and could be referred to the department of rheumatology of the Radboudumc or University Hospital Gent.

We adapted the VEDOSS criteria to assure that only patients with early disease could be included in the study. The following inclusion criteria were applied: age 18 years or older; presence of Raynaud’s phenomenon; positive test for associated auto-antibodies; early or active SSc pattern at nailfold capillaroscopy; and puffy fingers for <3 years. Main exclusion criteria were: any skin or internal organ involvement, including the presence of interstitial lung disease on high resolution CT-scan of the chest, previous treatment for SSc and anti-RNA polymerase III auto-antibodies because of the association between glucocorticoids and renal crisis, which is more prevalent in patients with anti-RNA polymerase III auto-antibodies (see Supplementary Table S1, available at *Rheumatology* online, for all eligibility criteria). All patients provided written informed consent.

### Treatment and procedures

Randomization was performed by an independent person from the hospital pharmacy of the Radboudumc. Patients were randomized in a 2:1 in randomly selected block sizes of 6, 9 and 12 or and stratified for gender, age (<40, 40–60, >60 years), and centre using Castor EDC (www.castoredc.com). A digital randomization list was kept at the hospital pharmacy of the Radboudumc.

Patients, nurses and physicians performing the assessments were blinded to the treatment allocation until the last patient had the 52-week follow-up visit and all the data were collected and locked in the database. At the 12-week assessment, the following close-ended question was asked to assess the success of blinding: ‘Which intervention group do you think you received, fake or treatment with methylprednisolone?’ A total of 28 of 30 patients (eight in the placebo group and 20 in the intervention group) guessed the allocation correctly.

Patients were assigned to receive either a daily infusion of methylprednisolone (1000 mg) or a placebo (physiological salt solution identical in appearance) on three consecutive days in three consecutive months. The study drug and placebo were provided by the local hospital pharmacy. Infusions were given at the outpatient facilities of both departments of rheumatology. All patients received an angiotensin-converting enzyme (ACE) inhibitor (enalapril) and a proton-pump inhibitor (pantoprazole) during the 12-week intervention period although evidence of the preventive use of an ACE inhibitor for renal crisis is debated. After the 12-week assessment, no further treatment was given, which is the standard care for early SSc. If signs and symptoms of disease progression became evident, the study medication was discontinued and treatment according to the EULAR treatment recommendations for SSc was started [26]. Disease progression was defined as the occurrence of any skin or internal organ involvement, such as significant interstitial lung disease, pulmonary arterial hypertension, (peri)myocarditis, renal crisis, gastric antral vascular ectasia, myositis, digital ulcers or pitting scars or tendon friction rubs. These criteria were developed on the basis of consensus among authors.

### Endpoints

The primary outcome was difference in NCM density between groups at week 12, evaluated as per EULAR consensus [27]. Differences in capillary density after 52 weeks, number of mega-capillaries, signs and symptoms of disease progression, time to disease progression, and patient-reported outcomes were secondary outcomes.

### Assessments

Patients were evaluated at the outpatient clinic including laboratory work up at baseline, 4, 8, 12, 24, 36 and 52 weeks. Pulmonary function tests were performed at screening, and at week 12, 24 and 52. Relevant pulmonary function decline after 1 year was defined as a ≥10% decrease in forced vital capacity (FVC) or a decrease in FVC ≥5% to <10% and a diffusing capacity of the lung for carbon monoxide (DLCO) decline of ≥15% [28]. High resolution CT and echocardiography were performed at screening and at week 52. Presence of interstitial lung disease was classified according to the GOH algorithm as limited or extensive [29].

Images of nailfold capillaries were acquired by three trained investigators (B.K., J.L., A.V.H.) using an optical probe videocapillaroscope equipped with a ×200 contact lens and connected to image analysis software (Videocap, DS MediGroup, Milano, Italy). The images were evaluated after the 52-week visit by two trained physicians (M.V., V.S.) who were blinded for the allocation. Two images per finger were evaluated and consisted of both quantitative and qualitative parameters. A total mean density score over all eight fingers was calculated. The modified Rodnan Skin Score was assessed as described before. The modified Rodnan Skin Score and other physical examinations were performed by trained assessors blinded for the intervention.

To assess daily functioning, gastrointestinal tract involvement and quality of life, patients were invited by mail to complete an online questionnaire (Castor EDC, Amsterdam, the Netherlands) consisting of the Scleroderma Health Assessment Questionnaire, Short Form-36 and Gastrointestinal Tract questionnaire at baseline, week 12 and 52 [30–33]. A maximum of two reminders were sent after 2 and 4 weeks.

Adverse events were collected at every patient contact until week 12 of follow-up by asking open questions. In addition, patients and their general practitioners were asked to report adverse events that occurred between two trial visits.

### Statistical analysis

We designed this study as a proof-of-concept study considering the lack of information of the effect of high-dose methylprednisolone on nailfold capillary changes and other outcomes in early SSc. We randomly assigned patients in a 2:1 ratio to enlarge the information about the experimental intervention, which could be justified as the risks of the intervention are known and relatively low. We therefore judged that inclusion of 30 patients (20 in the treatment arm and 10 in the placebo arm) would minimize the risk for patients, limit the recruitment time and be appropriate to inform future trials about the potential effect sizes of outcome measures. When adopting α of 0.05, power of 0.80, and a randomization scheme of 2:1, a sample size of 30 patients would be sufficient to detect a between-group difference of 1.0 in the capillary density score assuming a standard deviation of 0.9. We based our assumptions on a small study describing changes in nailfold capillaroscopic pattern after autologous stem cell transplantation treatment or cyclophosphamide treatment for diffuse cutaneous SSc. A statistically significant reduction of loss of capillaries, abnormal shapes (ramified capillaries) and capillary disorganization was only reported in patients treated with autologous stem cell transplantation, while in the group treated with cyclophosphamide no significant modifications were observed, these differences remained stable after 2 years of follow-up [22].

Descriptive statistics were provided as medians with interquartile range (IQR), or numbers with percentages (%) where appropriate. Analyses were performed using STATA 17 (StataCorp, College Station, TX, USA). All data were analysed according to the intention-to-treat principle. A *P*-value of 0.05 was used to denote statistical significance for between-group differences in the primary and secondary outcomes. Linear regression analyses adjusting for baseline values and stratification variables were performed to estimate differences between groups in continuous variables at 12 and 52 weeks. If applicable, the time to disease progression was calculated (see ‘Intervention’). A Kaplan–Meier curve, stratified by treatment, was constructed to describe disease progression over time. Adverse events regardless their relation with the intervention were categorized on the basis of consensus of two researchers (B.K., C.E.). No statistical analyses were done to compare adverse effects at 12 weeks. The study was funded by a legacy of a deceased SSc patient.

## Results

**Figure 1.**
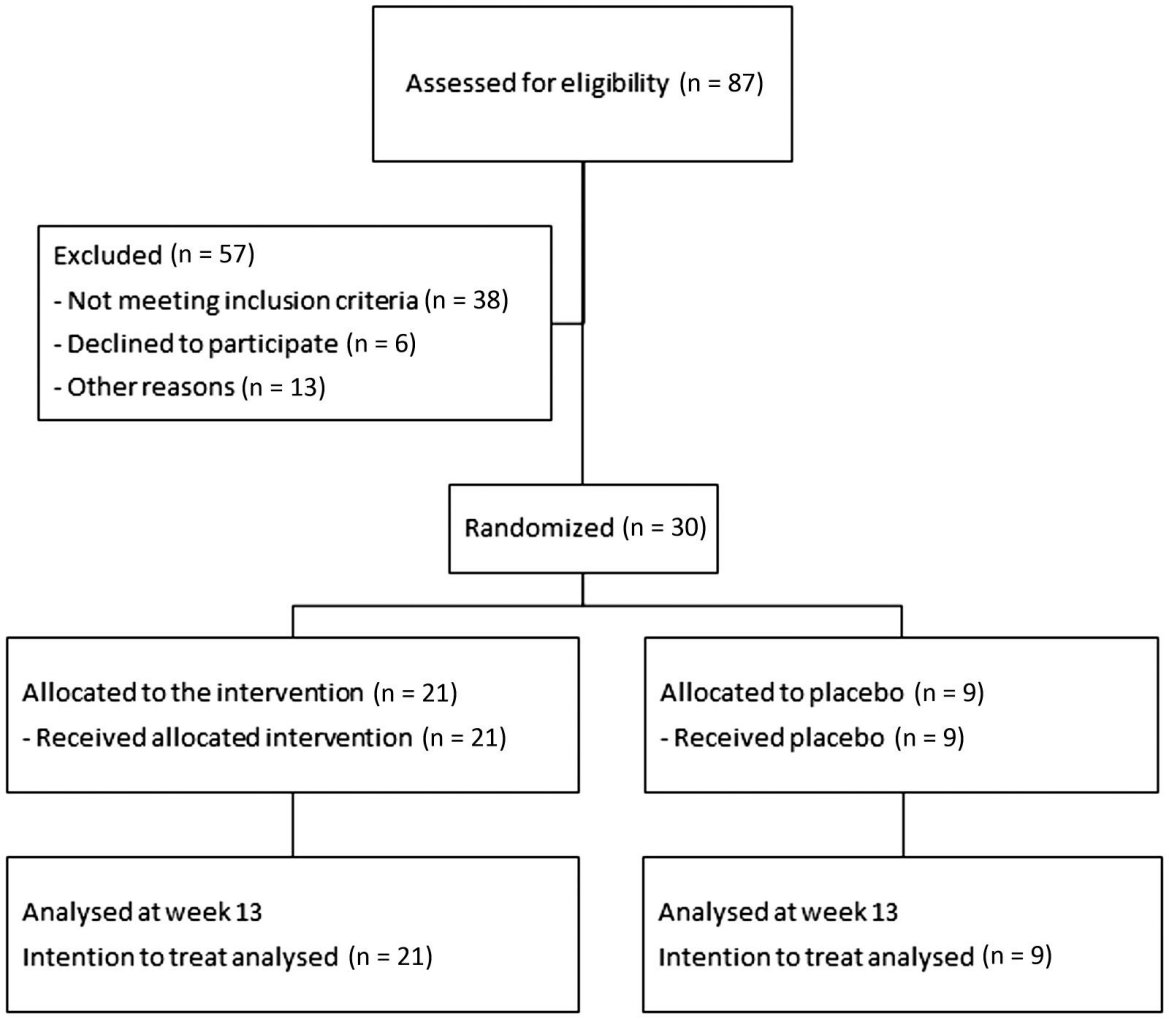
Flow-chart inclusion. Other reasons for not participating in the trial were disease progression or meeting exclusion criteria

Between February 2017 and February 2021, 87 patients were screened for eligibility. Fifty-seven patients (66%) of 87 were ineligible; most of them did not meet the inclusion criteria. Thirty individuals were included in the study, 21 (70%) were assigned to receive methylprednisolone, and nine (30%) individuals were assigned to receive placebo. All patients completed the 12-week assessment. Disease progression (skin involvement) in one patient in the methylprednisolone group at week 4 led to premature discontinuation of the intervention and initiation of standard of care. At week 52, 20 (95%) patients in the methylprednisolone group and eight (89%) in the placebo group completed the assessment (Fig. 1). One patient in the placebo group discontinued because of disease progression at week 26. The intention-to-treat and safety populations comprised nine patients in the placebo and 21 patients in the methylprednisolone group.

Baseline characteristics for the groups are presented in Table 1. Most patients were female (70%). The median (IQR) age was 52.9 (40.8–60.8) years. The median (IQR) duration from onset of first non-Raynaud feature to inclusion was 11.4 (4.6–18.6) months, 26 (87%) patients had puffy hands/fingers as first non-Raynaud’s phenomenon. Anticentromere antibodies were present in 22 (73%) patients. As for relevant previous history, one patient had breast cancer 20 years ago, two other patients had non-melanoma skin cancer. We did not observe differences between groups in the primary end point, −0.45 (95% CI: −1.11, 0.21) (Table 2).

**Table 1.** Baseline characteristics

Characteristic	Overall (30)	Placebo (*n* = 9)	Methylprednisolone (*n* = 21)
Age, median (IQR), years	52.9 (40.8–60.8)	53.3 (45.6–58.3)	52.6 (40.8–60.8)
Female, *n* (%)	21 (70)	7 (78)	14 (67)
Disease duration^a^, median (IQR), months	11.4 (4.6–18.6)	6.5 (4.6–17.3)	12.3 (5.8–18.8)
First non-Raynaud’s phenomenon			
Puffy hands, *n* (%)	26 (87)	7 (78)	19 (90)
Oesophageal involvement, *n* (%)	1 (3)	1 (11)	0 (0)
Telangiectasia, *n* (%)	2 (7)	0 (0)	1 (5)
Nailfold lesions by vision, *n* (%)	1 (3)	1 (11)	1 (5)
Puffy hands duration, median (IQR), months (*n* = 26)	12.2 (4.3–25.0)	11.6 (4.5–25.0)	13.0 (3.7–24.4)
Nailfold capillaroscopic pattern			
Early, *n* (%)	9 (25)	1 (11)	8 (32)
Active, *n* (%)	21 (75)	8 (89)	13 (68)
Normal capillary density^b^, *n* (%)	14 (47)	3 (33)	11 (52)
ESR, median (IQR), mm/h	7.0 (5.0–16.0)	5.0 (2.0–8.0)	8.0 (5.0–20.0)
Antibody			
Anti-centromere, *n* (%)	22 (73)	7 (78)	15 (71)
Anti-Scl70, *n* (%)	4 (13)	2 (22)	2 (10)
Anti-RNP, *n* (%)	3 (10)	0 (0)	3 (14)
Others^c^, *n* (%)	3 (10)	0 (0)	3 (10)
Pulmonary function			
FVC% predicted, median (IQR)	105 (95–117)	100 (95–112)	107 (98–118)
DLCO% predicted, median (IQR)	88 (81–99)	88 (81–95)	88 (81–99)
Open oesophagus^d^, *n* (%)	3 (10)	0 (0)	3 (14)
Current medication use			
Calcium channel blockers, *n* (%)	3 (10)	2 (22)	1 (5)
Angiotensin II receptor blockers, *n* (%)	0 (0)	0 (0)	0 (0)
ACE inhibitors, *n* (%)	2 (7)	0 (0)	2 (10)
Beta blockers, *n* (%)	1 (3)	0 (0)	1 (5)
5-HT2A antagonists, *n* (%)	1 (3)	0 (0)	1 (5)
Anticoagulants^e^, *n* (%)	3 (10)	0 (0)	1 (5)
Relevant medical history/comorbidity			
Malignancy, *n* (%)	3 (10)	0 (0)	3 (14)
Patient-reported outcomes			
SHAQ, median (IQR)	0.3 (0.0–09)	0.1(0.0–0.8)	0.2(0.0–0.9)
PCS SF-36, median (IQR)	41.5 (38.2–45.4)	41.6 (36.8–45.3)	41.5 (38.2–45.4)
MCS SF-36, median (IQR)	40.7 (33.9–46.0)	41.1 (32.6–45.9)	40.7 (33.9–46.0)
SSc–GIT, median (IQR)	0.2 (0.1–0.3)	0.1 (0.1–0.2)	0.2 (0.1–0.3)

a Disease duration from diagnosis to inclusion.

b Capillary density ≥7/mm.

c Other auto antibodies: anti-SM, anti-SSA and anti-fibrilarin.

d Oesophageal involvement on high resolution CT-scan.

e Anticoagulants: platelet agglutination inhibitor, adenosine diphosphate receptor inhibitors, selective factor Xa inhibitor, vitamine K antagonists.

ACE: angiontensin-converting enzyme; DLCO: diffusing capacity for carbon monoxide; FVC: forced vital capacity; SSc–GIT: SSc Gastrointestinal Tract survey; IQR: interquartile range; MCS SF-36: mental component scale short form health questionnaire; PCS SF-36: physical component scale short form health questionnaire 36; SHAQ: Scleroderma Health Assessment Questionnaire.

**Table 2.** Primary and secondary endpoints at week 12

	Placebo (*n* = 9)		Methylprednisolone (*n* = 21)		Difference between groups, mean (95% CI)^a^
	Baseline	Week 12	Baseline	Week 12	
Primary end point					
Capillary density, mm	7.0 (1.1)	7.3 (1.6)	7.3 (1.6)	7.2 (1.7)	−0.45 (−1.11, 0.21)
Secondary endpoints					
No. megacapillaries/mm	0.8 (0.7)	0.9 (0.8)	0.7(0.8)	0.6 (0.6)	−0.72 (−0.46, 0.11)
EULAR/ACR criteria,^b^n	10.7 (1.0)	11.2 (1.7)	10.1(1.2)	11.2 (2.9)	1.00 (−1.03, 3.03)

Values are presented as mean (s.d.) unless otherwise stated.

a Adjusted for baseline values and stratification variables.

b EULAR/ACR 2013 criteria [34].

In addition, no differences between groups in any of the secondary outcomes on disease characteristics (Supplementary Table S2, available at *Rheumatology* online) and self-reported outcome measures (i.e. Scleroderma Health Assessment Questionnaire [SHAQ], SF-36 and SSc Gastrointestinal Tract [SSc–GIT] survey; Table 3 and visual analogue scale; Supplementary Table S3, available at *Rheumatology* online) were found. We also did not observe a difference in puffy hands between baseline, week 12 and week 52. Only one patient in the intervention group did not have puffy hands at week 52. ESR did also not differ between groups and remained stable over time.

**Table 3.** Patient-reported outcome measures

	Placebo	Methylprednisolone						
	Baseline (*n* = 9)	Week 12 (*n* = 7)	Week 52 (n = 6)	Baseline (*n* = 19)	Week 12 (*n* = 15)	Week 52 (*n* = 16)	Difference at week 12, mean (95% CI) (*n* = 22)^a^	Difference at week 52, mean (95% CI) (*n* = 20)^a^
SHAQ	0.3 (0.4)	0.4 (0.4)	0.6 (0.5)	0.5 (0.5)	0.5 (0.6)	0.5 (0.6)	−0.04 (−0.28, 0.20)	−0.1 (−0.54, 0.40)
SSc–GIT	0.2 (0.1)	0.2 (0.2)	0.4 (0.5)	0.3 (0.3)	0.3 (0.3)	0.2 (0.3)	−0.5 (−0.25, 0.14)	−0.24 (−0.56, 0.08)
PCS SF36	40.9 (5.4)	40.0 (2.8)	40.3 (4.3)	41.2 (5.4)	40.2 (5.1)	42.7 (4.4)	−0.64 (−4.28, 3.0)	0.55 (−3.22, 4.31)
MCS SF36	39.8 (7.4)	41.6 (6.2)	39.8 (8.1)	40.5 (6.3)	38.7 (4.6)	39.0 (6.0)	−3.5 (−8.16, 1.17)	0.20 (−5.6, 6.0)

Values are presented as mean (s.d.) unless otherwise stated.

a Adjusted for baseline values and stratification variables.

SSc–GIT: SSc Gastrointestinal Tract survey; MCS SF-36: mental component scale short form health questionnaire; PCS SF-36: physical component scale short form health questionnaire 36; SHAQ: Scleroderma Health Assessment Questionnaire.

The total number of patients that fulfilled the definition of relevant pulmonary function decrease was 7 (23%): two in the placebo group and five in the methylprednisolone group at week 52. Of these patients none had significant interstitial lung disease on high resolution CT scan at week 52, according to the GOH algorithm [29].

**Figure 2.**
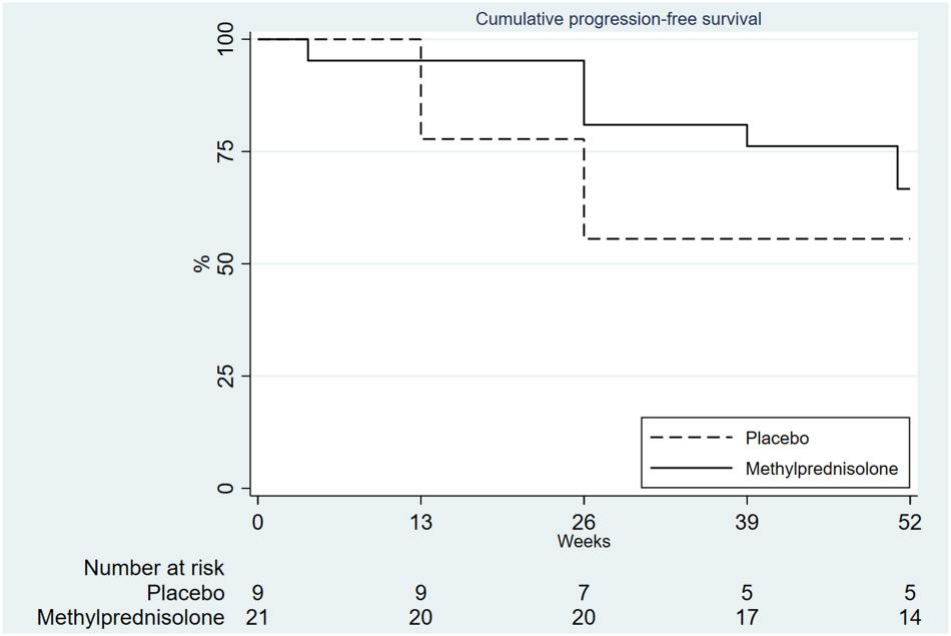
Kaplan–Meier curve showing time to disease progression. Blue line: placebo group; red line: methylprednisolone group

A total of 11 (37%) patients (seven in the methylprednisolone group and four in the placebo group) showed disease progression at week 52 (Fig. 2, Supplementary Table S4, available at *Rheumatology* online). Median (IQR) time to disease progression in these patients was 26 (12–39) weeks. Twenty seven percent of patients with ACA, 50% in anti-Scl 70 patients and 67% of patients with anti-RNP showed disease progression. One patient with anti-SM antibodies also progressed. In total, 14 patients (nine and five in the methylprednisolone and placebo group, respectively) showed either disease progression or a relevant decrease in pulmonary function.

Twenty-nine (97%) patients reported at least one adverse event but no serious adverse events were reported. Infections occurred in eight (38%) patients in the methylprednisolone group and in one (11%) patient in the placebo group. Infections requiring antibiotic treatment were urinary tract infections in two patients (10%) and pneumonia in three patients (14%). Medication-related adverse events were reported more frequently in the methylprednisolone group. Adverse events did not lead to withdrawals from the study (Table 4). Creatinine levels remained stable in both groups (74 µmol/l).

**Table 4.** Adverse events until week 12

	Placebo (*n* = 9)	Methylprednisolone (*n* = 21)
Patients with ≥1 adverse event, *n* (%)	8 (89)	21 (100)
Patients with ≥1 infectious adverse event, *n*	1	8
Patients with ≥1 serious adverse event, *n*	0	0
Medication related adverse events, *n*		
Psychological complaints	5	19
Neurological complaints	4	10
Intestinal complaints	4	8
Altered taste	1	10
Electrolyte imbalance	3	7
Endocrine system	5	25

## Discussion

We report data from the first ever investigator-initiated randomized, double blinded controlled trial in two European reference centres in SSc on treatment with methylprednisolone in very early SSc. There is insufficient evidence to confirm a clinically relevant treatment effect of methylprednisolone compared with placebo. Although the study is relatively small, all outcomes were consistent. However, more than one-third of the included patients showed disease progression after 12 months of follow-up.

We could not confirm our hypothesis that high dose methylprednisolone has a beneficial impact on the pathogenic vascular pathway of disease progression of SSc considering the lack of a beneficial clinical effect. There are possible explanations for this finding. First of all, our choice of the primary outcome measure, i.e. capillary density, could be questioned, considering the paucity of clinical data about changes in nailfold alterations after treatment. The available data on the value of NCM findings to date are all in patients with established disease including skin involvement. However, the value of NCM findings as a biomarker for SSc-associated vasculopathy has been intensely studied to date [35].

Secondly, the immunosuppressive and anti-inflammatory effect of methylprednisolone in very early SSc could be too small to detect in our relatively small study. However, the consistency of the lack of clinically relevant changes in all outcome measures and the observation that a considerable proportion of patients showed disease progression suggest that treatment with methylprednisolone does not result in a clinically important effect. Another reason might be that this adapted VEDOSS cohort, as it already includes patients with clinical symptoms (i.e. puffy fingers, telangiectasia) is not as ‘early’ as other ‘early’ cohorts. In line with this, a recent prospective follow-up of patients meeting LeRoy’s criteria (Raynauds, SSc-pattern on capillaroscopy and presence of SSc-specific antibodies) did not show a high progression in lung involvement over time [36, 37].

Thirdly, it may be hypothesized that a treatment duration of 3 months is insufficient to induce effects in very early patients. Finally, it is conceivable that methylprednisolone does not have an effect in this stage of the disease considering the hypothesis that the underlying pathogenic mechanism of SSc starts years before the first symptoms occur. The ineffectiveness of high dose glucocorticoids in very early SSc might challenge the hypothesis that inflammation is the initiating event in SSc or that interventions should be aimed at stages before clinical symptoms occur. Regarding safety, the adverse events that occurred were to be expected, renal crisis was not observed, and no serious adverse events or serious infections were reported.

To date, available criteria for very early SSc consist of the VEDOSS classification criteria, which combine Raynaud’s phenomenon, puffy fingers, positive antinuclear antibodies, and either SSc specific antibodies or scleroderma pattern NCM [16]. Applying the VEDOSS classification criteria does seem to result in a heterogeneous mixture of patients with early disease and patients with longstanding, very mild disease [21]. The follow-up of the VEDOSS study showed that 52.4% progressed to SSc after 5 years. However, this study is hampered by a low follow-up rate and lack of details about treatment of these patients during the 5 years of follow-up [20]. Koenig *et al.* reported that about 7 out of 10 patients from an ‘early’ cohort according to LeRoy (i.e. Raynaud’s phenomenon, disease specific antibodies and a SSc pattern on NCM) showed progression to SSc after 5 years of follow-up [38]. The actual risk for progression to SSc in patients with very early SSc during shorter follow-up has not been determined yet [20]. Therefore, to ensure that only patients early in the disease with risk for progression to SSc were included, we used an adapted version of the VEDOSS criteria by adding criteria, i.e. puffy fingers with a duration of < 3 years and the combination of scleroderma pattern on NCM and the presence of specific auto-antibodies. Our finding that about one-third of the patients indeed showed disease progression within 1 year indicates that the modification of the VEDOSS criteria might be useful both in clinical practice and in future research to select those patients for whom tight monitoring and early treatment is indicated. Considering that a substantial proportion of patients showed disease progression in only 1 year of follow-up, we believe there is a ‘window of opportunity’ for treatment in these patients. Treatment with other immunomodulating drugs than glucocorticoids should be explored in a follow-up study. Disease progression defined as the occurrence of any skin or internal organ involvement should be the primary end point. In addition, in our view a discussion among international experts to achieve a consensus-based decision about modification of the VEDOSS criteria will facilitate adoption of application of stricter criteria than the VEDOSS in research and clinical practice for this important category of patients.

This study is to our knowledge the first ever randomized controlled trial on treatment in very early SSc performed in two SSc European reference centres in SSc. Strengths of our study comprise the frequent standardized measurements of disease characteristics and patient-reported outcome measures. Measurements were performed by two experienced assessors, thus optimally reducing interobserver variability for skin involvement and assessment of nailfold alterations [27]. Follow-up was complete at week 12, the primary end point, and only two patients were lost to follow-up at week 52. Finally, we were able to select a homogeneous patient population at risk of disease progression.

The study has several limitations. First the relatively small sample size. This was chosen to maximize the information on this treatment and limit the recruitment time and possible adverse events. Second, blinding to the allocation was hampered because of known adverse events of methylprednisolone. Indeed, blinding was unsuccessful despite thorough precautions to conceal allocation, as the vast majority of patients guessed allocation correctly. Third, a number of patients did not complete the online questionnaires on patient-reported outcomes. Fourth, we used a rather complex randomization scheme with several strata and relatively large block sizes, which could have resulted in an imbalance in number of patients per group. However, we are convinced our results are not affected by the small imbalance in numbers of patients per group considering the resemblances of demographic and disease characteristics between groups. Fifth, we excluded anti-RNA polymerase III patients because of the heightened susceptibility to renal crisis. We wanted to minimize the risk of this serious adverse event in very early SSc patients. In principle, it is conjectured that such patients might benefit from glucocorticoids although we do not expect adding these patients would have influenced the results as all endpoints were negative.

In this investigator-initiated randomized double blind, placebo-controlled trial in patients with very early SSc, we were not able to find a positive effect of three monthly cycles of 3 days with methylprednisolone intravenously. We have shown that by adapting the VEDOSS criteria we were able to identify patients at risk of disease progression in 1 year. Future research should focus on conformation of this result and application of other immunomodulated treatments aiming to prevent progressive SSc.

## Supplementary Material

keae156_Supplementary_Data

## Data Availability

The data underlying this article will be shared on reasonable request to the corresponding author.
