# Supplementary material for: Efficacy of methylprednisolone in very early systemic sclerosis: results of the ‘Hit Hard and Early’ randomized controlled trial
Source: Rheumatology (Oxford). 2024 Mar 29;64(3):1261–9. doi: 10.1093/rheumatology/keae156 (PMC11879336; doi:10.1093/rheumatology/keae156)
Supplement: keae156_Supplementary_Data [file keae156_supplementary_data.docx]

Supplementary Table S1. In- and exclusion criteria

| Inclusion criteria | Exclusion criteria |
| --- | --- |
| - age 18 years or older - Raynaud’s phenomenon - SSc associated auto antibodies - nailfold capillaroscopic findings typical of SSc - puffy fingers for less than 3 years | - Acrosclerosis, acro-osteolysis or digital ulcers - Anti-RNA polymerase III auto-antibodies - Previous treatment with, methotrexate, prednisone (>14 days in the previous 6 months), mycophenolate mofetil or cyclophosphamide - Clinically significant internal organ involvement: diffusing capacity for carbon monoxide (DLCO) less than 80% predicted, vital capacity (VC) less than 70% predicted both caused by significant interstitial lung disease associated with SSc, renal dysfunction with glomerular filtration rate less than 60 mL/min, diastolic dysfunction more than grade 1, pulmonary hypertension, or weight loss of more than 10% in the last 6 months with unknown cause - Contra-indications for methylprednisolone |

Supplementary Table S2. Secondary endpoints at week 52.

|  | Placebo  (n=8) | | Methylprednisolone  (n=20) | | Difference at week 52  (mean, 95% CI) |
| --- | --- | --- | --- | --- | --- |
|  | Baseline | Week 52 | Baseline | Week 52 |  |
| Density, (mm) | 6.8 (1.1) | 6.5 (1.4) | 7.4 (1.6) | 7.0 (1.7) | -0.02 (-0.86, 0.81) |
| Number of mega capillaries | 0.9 (0.6) | 0.6 (0.5) | 0.6 (0.7) | 0.5 (0.5) | 0.06 (-0.27, 0.40) |
| EULAR / ACR criteria total score | 10.8 (1.0) | 11.4 (2.4) | 10.1 (1.1) | 11.6 (2.8) | -0.08 (-2.89, 2.73) |
| FVC predicted (%) | 105 (11) | 101 (7) | 105 (14) | 102 (14) | -0.1 (-5.9, 5.7) |
| DLCO predicted (%) | 91 (12)) | 86 (12) | 88 (13) | 85 (12) | 0.7 (-8.1, 9.6) |

Values are presented as mean (SD) unless otherwise stated. FVC: forced vital capacity. DLCO: diffusing capacity for carbon monoxide

Supplementary Table S3. Visual Analogue Scale.

|  | Placebo | | | Methylprednisolone | | |  |  | |  |  |
| --- | --- | --- | --- | --- | --- | --- | --- | --- | --- | --- | --- |
|  | Baseline  (n=8) | week 12  (n=7) | Week 52  (n=5) | Baseline  (n=19) | Week 12  (n=15) | Week 52  (n=16) | | | Difference at week 12 (mean, 95% CI)  (n=22) | | Difference at week 52 (mean, 95% CI)  (n=20) |
| VAS Pain | 37.5 (21.7) | 28.0 (22.1) | 41.0 (19.5) | 29.8 (26.8) | 29.4 (31.5) | 32.3 (28.5) | | | 2.29 (-12.38, 16.95) | | 1.56 (-17.61, 20.73) |
| VAS GI | 6.1 (8.4) | 4.4 (7.1) | 16.8 (17.4) | 4.7 (7.0) | 7.5 (10.6) | 3.3 (5.2) | | | 5.41 (- 2.81, 13.64) | | -12.65 (- 23.04 to -2.25) |
| VAS RP | 27.9 (13.0) | 21.6 (16.6) | 25.3 (12.2) | 32.9 (29.6) | 25.7 (28.4) | 29.9 (30.8) | | | -0.01 (- 20.69, 20.67) | | - 2.79 (-28.39, 22.8) |
| VAS DU | 15.3 (26.5) | 4.6 (9.9) | 4.2 (5.5) | 3.8 (14.4) | 5.1 (12.3) | 4.9 (12.5) | | | -0.41 (-7.33, 6.51) | | 1.01 (-1.81, 3.83) |
| VAS Severity | 32.9 (19.5) | 22.1 (18.9) | 32.8 (14.9) | 27.7 (26.5) | 27.5 (24.6) | 32.1 (28.7) | | | 7.14 (- 12.75, 27.03) | | 8.63 (-17.14, 34.4) |
| VAS Fatigue | 42.8 (33.4) | 41.3 (28.6) | 41.3 (29.7) | 42.9 (31.0) | 45.1 (26.3) | 37.8 (34.1) | | | 8.85 (-9.08, 26.77) | | 9.42 (-10.21, 29.05) |

Values are presented as mean (SD) unless otherwise stated. VAS: Visual Analogue Scale. VAS measured on different disease domains. GI: gastroinstestinal, RP: Raynauds phenomenon, DU: digital ulcera.

Supplementary Table S4: Number of patients with signs of disease progression observed during first year of follow up

|  | Placebo  (n=9) | Methylprednisolone  (n=21) | Total  (n=30) |
| --- | --- | --- | --- |
| Skinprogression * | 2 | 4 | 6 |
| Digital ulcera or pitting scars | 3 | 3 | 6 |
| Gastric antral vascular ectasia | 1 | 1 | 2 |
| Pulmonary arterial hypertension | 0 | 1 | 1 |
| N patients with ≥ 1 sign of progression | 4 | 7 | 11 |
| Antibody**:   - ACA (n, %) - ATA (n, %) - Anti RNP (n, %) - Anti SM (n, %) |  |  | 6 (27%)  2 (50%)  2 (67%)  1 (100%) |

*Defined as modified Rodnan skin score > 0 after baseline

** Antibody profile of patients showing disease progression
